# Prediction and Characterization of Glycated Peptides and Proteins using Hydrophilic Interaction Liquid Chromatography coupled with Mass Spectrometry (HILIC-MS)

**DOI:** 10.7171/001c.157968

**Published:** 2026-03-20

**Authors:** Sonal Priya, Elizabeth L Kowalski, Marla Popov, Ron Orlando

**Affiliations:** 1 Complex Carbohydrate Research Center University of Georgia; 2 Department of Chemisty University of Georgia; 3 Chemistry and Biochemistry California State Polytechnic University; 4 Research GlycoScientific (United States)

**Keywords:** Liquid chromatography–mass spectrometry, Hydrophilic Interaction Liquid Chromatography (HILIC), Glycation, Separation, mAbs, monoclonal antibodies, Biopharmaceuticals, Retention Prediction

## Abstract

Glycation is an important post-translational modification (PTM) that has been linked to diabetes, cataract, Alzheimer’s, and Rheumatoid arthritis. This reaction occurs between a reducing sugar and a primary amine at the N-terminus of a protein or at a lysine side chain. Ultimately, this interaction can lead to advanced glycation end products (AGEs) that are associated with several disease complications. Glycation can occur during the manufacturing and storage of therapeutic proteins, including monoclonal antibodies (mAbs), necessitating the characterization of this modification to ensure the safety and efficacy of therapeutic drug products. Hydrophilic Interaction Liquid Chromatography (HILIC) has been previously employed to characterize hydrophilic modifications. It can also be used to characterize glycated species, as the hydrophilic nature of the glycation product can lead to a characteristic shift in HILIC retention. This work focuses on deriving a retention coefficient that describes the extent of hydrophilicity imparted by glycation modification in HILIC using in vitro glycated peptide and protein samples. The HILIC retention coefficient can be used to predict the retention times of tryptic peptides with glycation modifications in complex, unknown protein samples, including immunoglobulins (IgGs).

## Introduction

The demand for biopharmaceuticals has significantly grown in recent years, with a global market size of $448.1 billion in 2023 and an expected increase of 7.6% (CAGR) between 2024 and 2030 to reach $745.1 billion by 2030.^1^ One of the key challenges that drug developers and manufacturers need to combat is ensuring the drug product’s quality, efficacy, and safety. Most biological drugs are proteins that can undergo post-translational modifications (PTMs), which can affect their biological activity, immunogenicity, and half-life.^2,3^ Common protein modifications include glycosylation, oxidation, deamidation, and glycation, among others. Characterizing these modifications can be a complicated and time-consuming part of the biotechnology industry.

**Figure 1.**
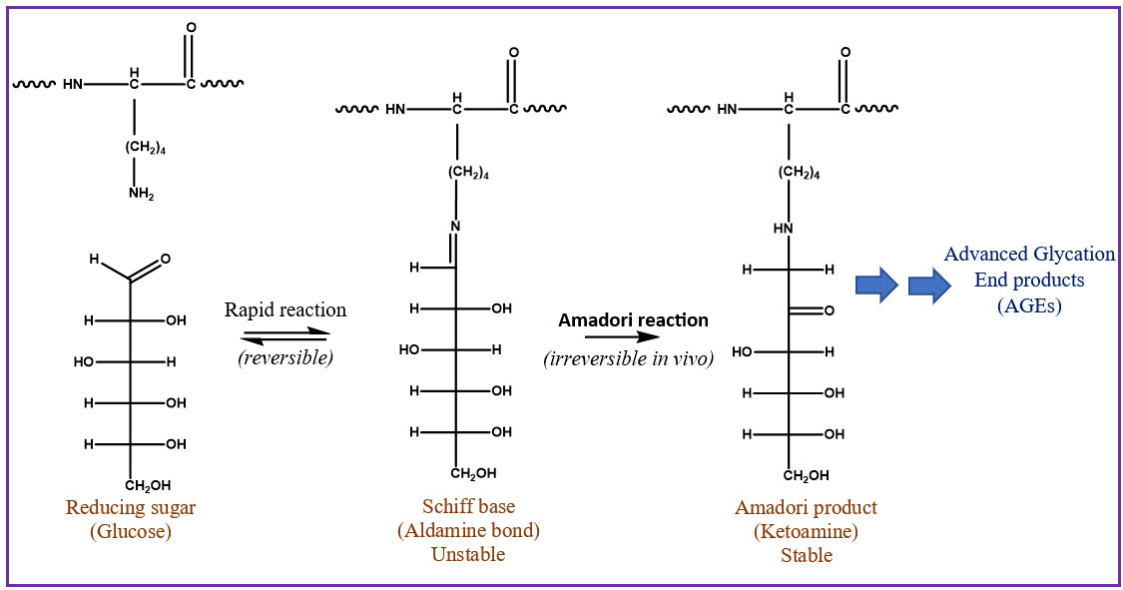
The Scheme for Glycation Reaction on a Lysine Residue. The reducing end of sugar can undergo Schiff’s base reaction with the primary amine of lysine residue to produce a ketoamine Amadori product. The Amadori product, in the presence of reactive intermediates, can ultimately lead to advanced glycation end products (AGEs).

Glycation is an important PTM linked to diabetes, Alzheimer’s, cataract formation, and rheumatoid arthritis.^4,5^ This process involves a reaction between a reducing sugar, such as glucose, fructose, or galactose, and the alpha amine terminal of a protein or epsilon amine group on a lysine side chain.^6^ An unstable Schiff’s base intermediate is produced that can potentially undergo a spontaneous multistep Amadori rearrangement, giving rise to a more stable, covalently bonded ketoamine product (Figure 1). Glycated proteins, when exposed to elevated oxidation conditions over a prolonged period, can undergo further degradation, resulting in advanced glycation end products (AGEs). AGEs could be responsible for various pathological responses observed in aging, diabetes, and arthritis caused by intracellular accumulation, cross-linking with tissue macromolecules, and interactions with a specific receptor for AGEs called RAGE.^7–9^

Glycation is a potential critical quality attribute (CQA) in therapeutic monoclonal antibodies (mAbs). This PTM could be introduced during the fermentation step where glucose acts as an energy source for mAb-producing cells.^10^ Glycation could also be introduced during the storage of therapeutic mAbs due to the presence of carbohydrates in the formulation.^11^ Although nonreducing disaccharides, such as sucrose, are typically used in the formulation, sucrose hydrolysis could produce reducing sugars under acidic pH and elevated temperatures, thereby leading to glycation. In addition, glucose is an ingredient in commonly used drug infusion solutions. Glycation can alter the overall charge on therapeutic proteins, thereby hindering their biological function and reducing their bioactivity.^12^ Antibody aggregation is also an important CQA associated with AGE formation and cross-linking.^11^ AGE-damaged antibodies could also trigger an immune response by forming anti-IgG autoantibodies in patients with rheumatoid arthritis. It has been demonstrated that glycation reduces the stability and half-life of apolipoprotein I (ApoAI) in humans.^13^ These findings suggest that glycation may have similar effects on other proteins.

Some challenges associated with glycation characterization include the absence of a specific sequence that signals a potential glycation site, given that the reaction is nonenzymatic.^14^ If a protein does not contain a highly reactive site for this modification, glycation can occur across the entire protein, which results in low levels of glycation on all the susceptible glycation sites and leads to a heterogeneous mixture of many low-abundance species.^15^ Therefore, it is essential to perform comprehensive studies to characterize glycated products to ensure the safety and efficacy of therapeutic drugs.

One of the current strategies used for glycation characterization is boronate affinity chromatography (BAC),^10,16^ which requires native running conditions and minimal sample preparation. However, nonspecific binding can occur as nonglycated species can also interact with the BAC surface. In addition, quantification is challenging because it is difficult to distinguish between proteins with a single glycation versus multiple glycations.^17^ Another strategy to identify glycated species is based on the molecule’s charge, as glycation neutralizes the positive charge at the glycation site. Therefore, charge-based methods such as capillary isoelectric focusing (cIEF),^17^ imaged capillary isoelectric focusing (icIEF), and ion-exchange chromatography (IEC) can be employed.^10^ IEC may lack sufficient resolution to resolve the glycated variants due to combined charge effects arising from multiple sites of low-level glycation spread across the molecule. Charged sites resulting from different PTMs could further complicate the analysis of complex protein samples, such as antibodies.

Liquid chromatography–mass spectrometry (LC-MS) has been widely utilized for efficient characterization of PTMs.^17^ Top-down mass spectrometry of intact antibody or enzymatically cleaved monoclonal antibody fragments can be used to determine the glycation level as each glycation event results in a 162 Da mass increase. Bottom-up peptide mapping is widely used to locate glycation sites.^12,14^ Glycation can inhibit trypsin activity at the C-terminus of lysine residues. Thus, a missed trypsin cleavage at a lysine residue, with a +162 Da shift in MS, indicates a glycated peptide.^17,18^ Furthermore, HILIC separation mode is an effective strategy to characterize hydrophilic modifications such as deamidation, methionine oxidation, and glycosylation.^18–21^ Glycation involves the addition of a hydrophilic glucose molecule, which may increase HILIC retention. Glycation PTM also neutralizes the positive charge on primary amines, which could decrease hydrophilicity and thus decrease HILIC retention time. It was hypothesized that a combined effect from these two circumstances could result in a characteristic shift under HILIC mode.

This study was undertaken to develop a robust analytical workflow to characterize glycation sites in therapeutic mAbs. Retention behavior of several in vitro glycated standard peptides and proteins on HILIC was studied, and a retention coefficient for this modification was derived using dextran as an external calibrant. This retention coefficient, in combination with an existing model^20^ predicting the HILIC retention of tryptic peptides, was used to predict and characterize glycation in unknown mAbs, including human IgG1 (adalimumab) and IgG4 (natalizumab). Model fit was evaluated using these test cases.

## Materials and Methods

### Protein Digestion

Protein samples, including bovine insulin and bovine cytochrome C (both purchased from Sigma Aldrich, St. Louis, MO, USA), human IgG1 (adalimumab), and IgG4 (natalizumab) from GlycoScientific (Athens, GA, USA), were buffer exchanged with 50 mM ammonium bicarbonate (pH 7.8) to have a final concentration of 1 mg/mL. The protein samples were then reduced with 200 mM dithiothreitol (DTT) and alkylated with 1 M iodoacetamide (Sigma Aldrich), both at final concentrations of 5 mM DTT and 8 mM iodoacetamide. Sequencing-grade trypsin from Promega (Madison, WI, USA) was added at a 20:1 (w/w, protein:trypsin) ratio and incubated at 37°C overnight. The digested protein samples were then dried in a SpeedVac and resuspended in 80% ACN (1 mg/mL) and 20% H_2_O prior to LC-MS analysis. Endoproteinase Glu-C (*Staphylococcus aureus* strain V8; Thomas Scientific, Swedesboro, NJ, USA) digestion of human IgG1 was performed as previously mentioned, with the similarly previously discussed reduction and alkylation, and Glu-C was then added at a final protein:proteinase ratio of 20:1 (w/w). IgG1 was then incubated overnight at 37°C in either PBS or ammonium bicarbonate.

### In Vitro Glycation

A series of standard peptides, such as human [Glu1]-Fibrinopeptide B (GluFib), leucine enkephalin, bradykinin, and substance P (Frag 2-11), were purchased from Sigma Aldrich. The standard peptides, along with digested bovine cytochrome C, bovine insulin, human IgG1, and IgG4, were glycated in vitro. GluFib, leucine enkephalin, bradykinin, substance P, insulin, and cytochrome C were glycated using a 2:1 D-glucose:peptide/protein molar ratio at a pH of 2.4 (65°C, 1 day). A D-glucose:protein molar ratio of 1000:1 was used for IgG samples. The samples were then dried and resuspended in 80% ACN (1 mg/mL) for LC-MS analyses.

Procainamide-labeled dextran (M_r_ 6,000) from Sigma Aldrich was used as a retention calibrant, and procainamide-labeled maltopentaose from Supelco Analytical (Bellefonte, PA, USA) was used as an internal standard. For 200 μg each of dextran and maltopentaose, 60 µL of 0.4 M procainamide was added. HCl (Sigma Aldrich) and 0.8 M sodium cyanoborohydride (Sigma Aldrich) were mixed in dimethyl sulfoxide (DMSO):acetic acid at a ratio of 7:3 (v/v) and incubated at 65°C overnight. The samples were dried using a SpeedVac, resuspended in 5% acetic acid (240 μL), and cleaned on a PD MiniTrap G10 desalting column (Cytiva, Marlborough, MA, USA) according to the manufacturer’s protocol. The samples were then dried and resuspended in 80% ACN (1 mg/mL).

### LC-MS Settings and Instrumentation

Data were acquired using an Agilent 1100 series (Santa Clara, CA, USA) coupled to a Waters SYNAPT-G2 QTOF (Milford, MA, USA) system with an electrospray ionization (ESI) source operated in positive-ion mode. Peptides were separated using a 2.1-mm × 150-mm HALO Penta-HILIC column packed with 2.7-μm diameter superficially porous particles with a 90-Å pore diameter (Advanced Materials Technology, Wilmington, DE, USA) at 60°C column temperature. The solvents used for separation were 50 mM ammonium formate in water with 0.1% formic acid (Solvent A) and 0.1% formic acid in Acetonitrile (Solvent B). A linear gradient ranging from 80% to 40% Solvent B over 40 minutes (1% B per minute) at 0.2 mL/minute flow rate was used for separation. MS data were acquired in full-scan MS over a m/z range suitable for peptide detection (300–2000 m/z). The ESI source was operated at a capillary voltage of 3.0 kV, a sampling cone voltage of 20 V, an extraction cone voltage of 3.0 V, and a source temperature of 120°C. Analysis of mass spectral data was performed using Waters MassLynx, ProteinLynx Global Server, and Skyline software. Extracted ion chromatograms (XICs) were generated using a mass width of ±100 ppm. Peptide retention times in minutes were converted to glucose units based on dextran samples that were run immediately before and after the protein/peptide sample of interest.

## Results and Discussion

### Resolving Glycated Peptides from Unmodified Peptides

**Figure 2.**
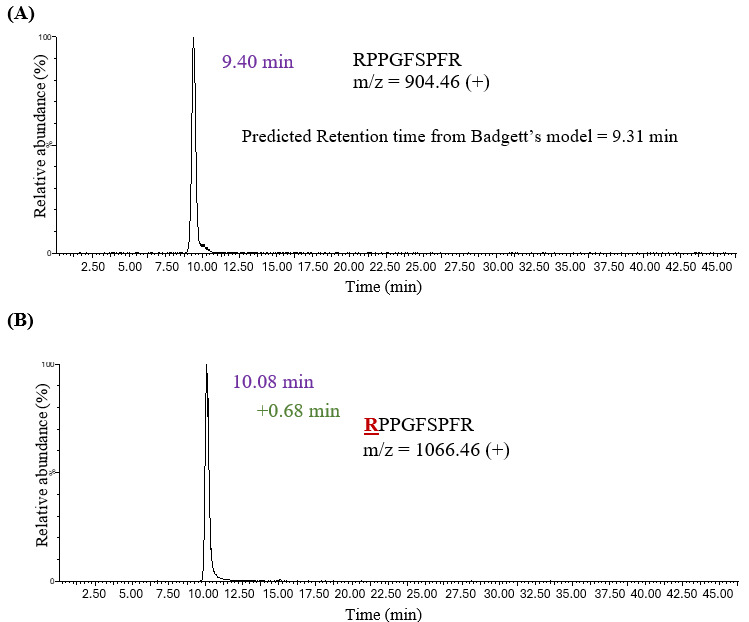
The XIC for (A) Unmodified Bradykinin RPPGFSPFR and (B) Glycated Bradykinin RPPGFSPFR, Demonstrating a Net Increase in HILIC Retention (+0.68 Minutes) Upon Glycation. The experimental retention time of the unmodified peptide (9.40 minutes) was observed to be close to the retention time predicted by Badgett’s model (9.31 minutes).

The study began with in vitro glycation^22^ of carefully selected standard peptides that possess only a single glycation site, using the low pH conditions described in the Methods section. Peptides, including human [Glu1]-fibrinopeptide B (GluFib), leucine enkephalin, bradykinin, and substance P (Frag 2-11), were chosen. Substance P (Frag 2-11) was used to study lysine glycation as it contains proline at the N-terminus with no primary amine available for glycation at that site. The in vitro glycated samples were run on a Penta-HILIC column to examine the retention behavior of the glycated variants. Badgett’s retention model^20^ was first used to confirm the peaks corresponding to the unmodified peptides. In this instance, the experimental retention time of unmodified bradykinin (RPPGFSPFR) was 9.40 minutes (Figure 2), which was close to the retention time predicted by Badgett’s model (9.31 minutes).

The experimental retention times of all unmodified peptides correlated well with their predicted retention times and were calculated using retention parameters from Badgett’s model. A single glycated peptide with +162 Da m/z was observed for each standard peptide. For example, glycated Bradykinin (RPPGFSPFR) was eluted at 10.08 minutes, demonstrating a net increase in HILIC retention (+0.68 minutes) upon glycation. Thus, a characteristic chromatographic shift was observed for the glycated variant in HILIC mode relative to its unmodified counterpart. The retention shift ranged from +0.21 minutes for leucine enkephalin to +1.19 minutes for Substance P (Table 1). Glycation introduces a hydrophilic glucose moiety that can increase retention on a HILIC column, but this modification also neutralizes the positive charge at the primary amine, thus decreasing hydrophilicity. A combined effect of both factors resulted in a net increase in retention time upon glycation.

**Table 1.** Calculation of HILIC retention coefficient for glycation PTM using standard peptides and proteins. The average glycation coefficient was calculated to be +0.35 Glucose Units (GU) with a standard deviation of 0.12 GU

**Protein/Peptide**	**Sequence**	**Retention Shift Upon Glycation (Minutes)**	**Retention Shift Upon Glycation (GU)**
Cytochrome C (bovine)	TGQAPGFSYTDAN**K**NK	+0.22	+0.14
	H**K**TGPNLHGLFGR	+0.28	+0.18
	EDLIAYL**K**K	+0.31	+0.19
	**K**TGQAPGFSYTDANK	+0.59	+0.31
	**K**IFVQK	+0.71	+0.40
	MIFAGI**K**K	+0.72	+0.41
	GITWGEETLMEYLENP**K**K	+0.78	+0.46
	**K**YIPGTK	+0.79	+0.46
	YIPGT**K**MIFAGIK	+1.15	+0.53
	YIPGT**K**MIFAGIK	+1.04	+0.51
	MIFAGI**K**K	+0.45	+0.24
Insulin (bovine)	**G**IVEQCCASVCSLYQLENYCN	+0.77	+0.49
	**F**VNQHLCGSHLVEALYLVCGERGFFYTPKA	+0.73	+0.45
GluFib	**E**GVNDNEEGFFSAR	+0.46	+0.24
Bradykinin	**R**PPGFSPFR	+0.68	+0.35
Leucine enkephalin	**Y**GGFL	+0.21	+0.12
Substance P (Frag 2-11)	P**K**PQQFFGLM	+1.19	+0.54
*Average coefficient for glycation*		***+0.35 GU***	
*Standard deviation of glycation*		*0.12 GU*	

Proteins containing more than one glycation site were analyzed next, including bovine insulin and bovine cytochrome C. Glycation sites were identified based on the peptide sequence, trypsin digestion patterns, mass shifts corresponding to glucose addition, and chromatographic behavior under HILIC conditions. Nonenzymatic glycation targets nucleophilic amino groups, (i.e., the N-terminal α-amino group and the ε-amino group of lysine residues). Glycation at internal lysine residues was inferred based on the presence of missed tryptic cleavage sites, which is consistent with modification of the lysine side chain. Glycation at the N-terminus can only occur at the original N-terminus of the peptide/protein as the glycation was carried out prior to trypsin digestion. Glycation at the N-terminus was further supported if the peptide lacked an internal lysine residue. Together, these criteria provide a basis for assigning glycation sites to either N-terminal amino groups or lysine side chains within the identified peptides.

LC-MS analysis after S-S cleavage in insulin revealed an increase in retention time for N-terminus glycated chain A (+0.77 minutes) and chain B (+0.73 minutes) peptides when compared to their unmodified counterparts (Table 1). In addition, in vitro glycation was performed on bovine cytochrome C, followed by trypsin digestion. Cytochrome C has 18 lysine residues that could undergo glycation. There were 11 peptides identified using MS1 spectra glycated at the lysine residues. Glycation on the lysine side chain generated a “missed cleavage” peptide with an additional 162 Da m/z. Trypsin has a negatively charged pocket that confers specificity for amino acids with positively charged side chains (i.e., lysine and arginine). The addition of sugar to the lysine side chain neutralizes its charge and increases its size, thereby reducing enzymatic activity at glycation sites. Therefore, a missed tryptic cleavage peptide, in combination with a +162 Da m/z shift, is used as an indicator of glycation. The retention shift for lysine glycation was calculated relative to the unmodified missed cleavage peptide. The experimental retention times for the unmodified variants were consistent with those predicted by Badgett’s model.

**Figure 3.**
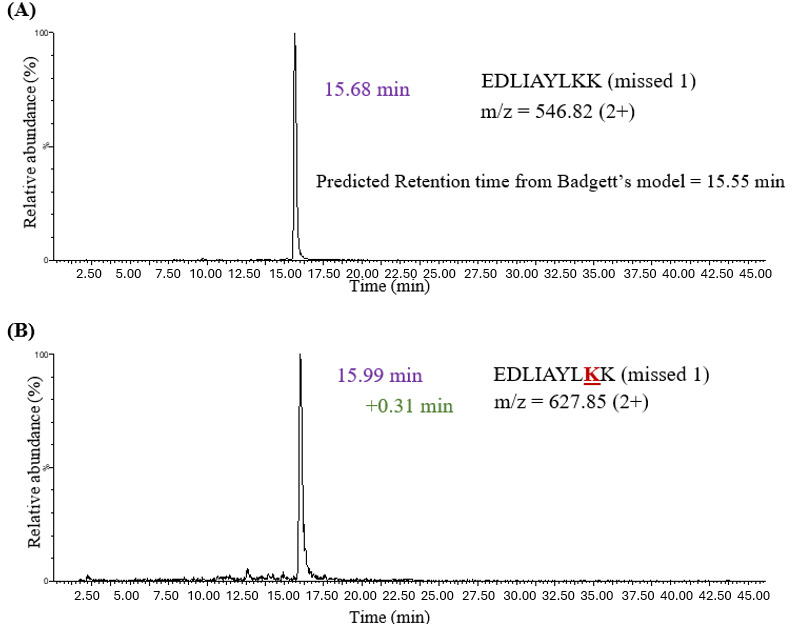
The XIC for (A) Unmodified Missed Cleavage Peptide EDLIAYLKK and (B) Glycated Missed Cleavage Peptide EDLIAYLKK in Bovine Cytochrome C, Demonstrating an Overall Increase in HILIC Retention (+0.31 Minutes) upon Glycation. The experimental retention time of the unmodified peptide (15.68 minutes) was observed to be close to the retention time predicted by Badgett’s model (15.55 minutes).

Figure 3 demonstrates that the unmodified EDLIAYLKK peptide in cytochrome C with one missed cleavage eluted at 15.68 minutes, which was close to the retention time calculated by Badgett’s model (15.55 minutes). The glycated missed cleavage peptide EDLIAYLKK demonstrated an overall increase in HILIC retention (+0.31 minutes) upon glycation. Retention time shifts for glycated peptides ranged from +0.22 minutes to +1.15 minutes (Table 1). In all cases, glycation increased retention time, regardless of whether the modification occurred at the N-terminus or on a lysine side chain. However, the magnitude of the shift varied with the peptide sequence and the site of modification.

### Derivation of HILIC Retention Coefficient

The retention shifts have been expressed in minutes thus far. To use this prediction model on any LC-MS system, a retention calibrant consisting of procainamide-labeled dextran was required. Procainamide-labeled dextran samples were run before and after the glycated protein/peptide sample; the retention times were averaged and used to convert peptide retention times in minutes to glucose units (GU) based on a logarithmic fit to the dextran samples.^20^ The retention time difference (in minutes) between a glycated and nonglycated peptide was therefore converted to GU and used to calculate the HILIC retention coefficient for this modification in GU. This strategy enables the model to be applied to any LC-MS system and any gradient, provided that the dextran standard is run under the same separation conditions as the protein sample of interest. The HILIC retention coefficient for glycation PTM from standard peptides and proteins was calculated to be +0.35 GU with a standard deviation of 0.12 GU (Table 1).

### Prediction of Glycation in Unknown Test Samples

**Figure 4.**
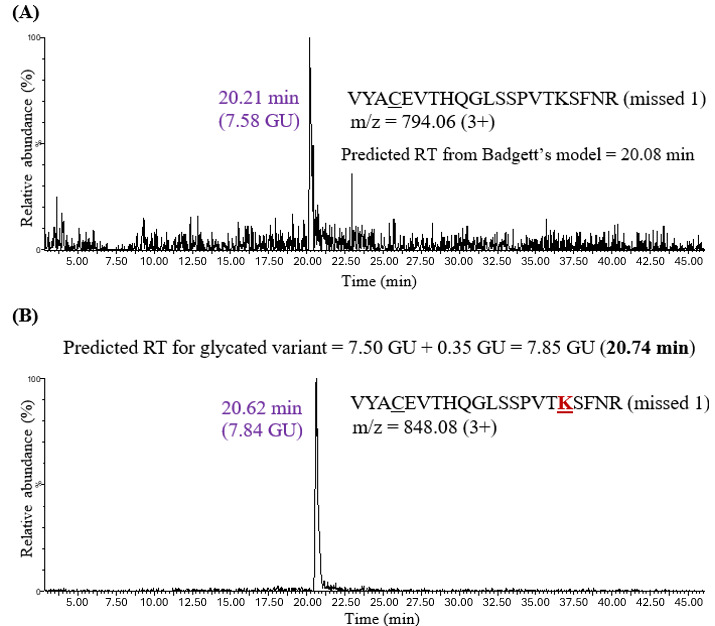
The XIC for (A) Unmodified Missed Cleavage Peptide VYACEVTHQGLSSPVTKSFNR and (B) Glycated Missed Cleavage Peptide VYACEVTHQGLSSPVTKSFNR in Human IgG1 (Adalimumab). The experimental retention time for the unmodified peptide (20.21 minutes) was observed to be close to the retention time predicted by Badgett’s model (20.08 minutes). The experimental retention time of the glycated variant (20.62 minutes) was close to the predicted retention time (20.74 minutes), calculated from the HILIC retention coefficient for glycation.

The HILIC retention model for glycation PTM was tested by predicting and characterizing glycation in more complex IgG samples. Adalimumab glycation was achieved by incubating the protein at 65°C for one day, followed by reduction, alkylation, and overnight trypsin digestion. Glycation can occur at the N-termini of light and heavy chains as well as on lysine residues. The predicted retention time for the glycated peptide was calculated by adding 0.35 GU (coefficient derived for glycation) to Badgett’s predicted retention time for the unmodified peptide (in GU). For instance, the predicted retention time of the VYACEVTHQGLSSPVTKSFNR missed cleavage peptide, according to Badgett’s model, was 7.50 GU, which corresponds to 20.08 minutes (Figure 4).

**Figure 5.**
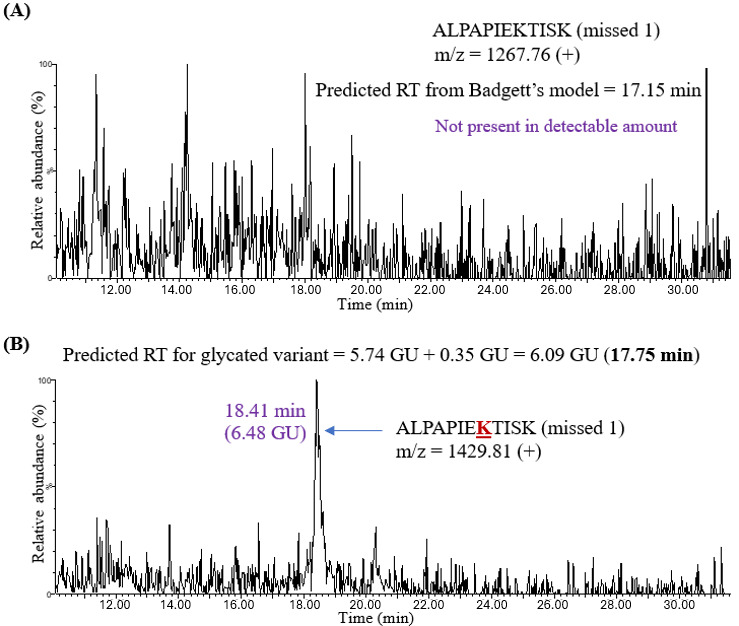
The XIC for (A) Unmodified Missed Cleavage Peptide ALPAPIEKTISK and (B) Glycated Missed Cleavage Peptide ALPAPIEKTISK in Human IgG1 (Adalimumab). The predicted retention time of the unmodified peptide calculated using Badgett’s model was 17.15 minutes, but the unmodified peptide was not present in a detectable amount. The experimental retention time of the glycated variant (18.41 minutes) was close to the predicted retention time of 17.75 minutes, which was calculated using the HILIC retention coefficient for glycation.

The experimental retention time for this peptide was 20.21 minutes (7.58 GU), which was close to the predicted value. The retention time of its glycated variant VYACEVTHQGLSSPVTKSFNR was then predicted by adding 0.35 GU to 7.50 GU, which gave 7.85 GU or 20.74 minutes. A chromatographic peak at 20.62 minutes (7.84 GU) was observed, close to the predicted retention time. It is worth noting that the peak intensity of the unmodified missed cleavage peptide was low, which may be due to trypsin efficiently cleaving at the C-terminus of every lysine and arginine residue. The unmodified missed cleavage peptides were not detected in all the cases (Figure 5A), which highlights the importance of calculating a predicted retention time for glycation by adding 0.35 GU to Badgett’s predicted retention time (in GU), which was developed for unmodified variants.

The unmodified peptide ALPAPIEKTISK (missed 1), for example, was not detectable (Figure 5A). Even so, the predicted retention time of glycated ALPAPIEKTISK (missed 1) could be calculated by adding 0.35 GU to Badgett’s predicted retention time for the unmodified peptide, (i.e., 5.74 GU, resulting in 6.09 GU or 17.75 minutes in Table 2). A peak at 18.41 minutes was observed in the XIC corresponding to the m/z of singly charged ALPAPIEKTISK peptide (Figure 5B). Thus, the experimental retention times for glycated variants were close to the predicted retention times. The predicted and experimental retention times for all detected unmodified and glycated peptides are presented in minutes and GU in Table 2. Overall, the average retention shift in adalimumab tryptic peptides upon glycation was +0.50 GU, which is reasonably close to the coefficient of +0.35 GU determined from the analysis of standard peptides and proteins.

**Table 2.** Prediction and characterization of glycation in a human IgG1 sample (Adalimumab). The experimental retention time of glycated peptides was close to the predicted value. Average retention shift upon glycation was observed to be +0.50 GU. The experimental retention times of the unmodified peptides (in minutes and in GU) are also listed when detected

**IgG1 Peptide Sequence**	**Predicted RT (GU)**	**Predicted RT (min)**	**Experimental RT (min)**	**Experimental RT (GU)**	**Retention Shift upon Glycation** **(min)**	**Retention Shift upon Glycation (GU)**
ALPAPIEKTISK	5.74	17.15				
ALPAPIE**K**TISK	6.09	17.75	18.41	6.48	+1.26	+0.74
LTVDKSR	5.87	17.37	18.85	6.74		
LTVD**K**SR	6.22	17.97	19.52	7.15	+1.15	+0.27
EVQLVESGGGLVQPGR	5.09	15.95	16.68	5.48		
**E**VQLVESGGGLVQPGR	5.44	16.61	17.47	5.93	+1.52	+0.64
VVSVLTVLHQDWLNGKEYK	5.91	17.44				
VVSVLTVLHQDWLNG**K**EYK	6.26	18.04	17.64	6.02	+0.20	+0.11
VSNKALPAPIEK	6.31	18.13				
VSN**K**ALPAPIEK	6.66	18.71	20.31	7.64	+1.18	+0.83
DELTKNQVSLTCLVK	6.94	19.17				
DELT**K**NQVSLTCLVK	7.29	19.74	19.67	7.24	+0.50	+0.30
APKLLIYAASTLQSGVPSR	4.78	15.33	16.63	5.45		
AP**K**LLIYAASTLQSGVPSR	5.13	16.02	16.42	5.38	+1.09	+0.60
APYTFGQGTKVEIK	6.35	18.20	18.51	6.54		
APYTFGQGT**K**VEIK	6.70	18.77	18.92	6.79	+0.72	+0.43
VYACEVTHQGLSSPVTKSFNR	7.50	20.08	20.21	7.58		
VYACEVTHQGLSSPVT**K**SFNR	7.85	20.74	20.62	7.84	+0.54	+0.34
DIQMTQSPSSLSASVGDR	6.83	18.99				
**D**IQMTQSPSSLSASVGDR	7.18	19.57	20.22	7.58	+1.23	+0.75
ADYEKHK	8.59	21.73				
ADYE**K**HK	8.94	22.16	22.44	9.12	+0.71	+0.53

All the glycated peptides were confirmed using the retention model and MS1 spectra. Glycated peptides have low ion currents, making their analysis by MS/MS challenging as the chance that the ion corresponding to the glycated variant would be selected for MS2 was very low.

**Figure 6.**
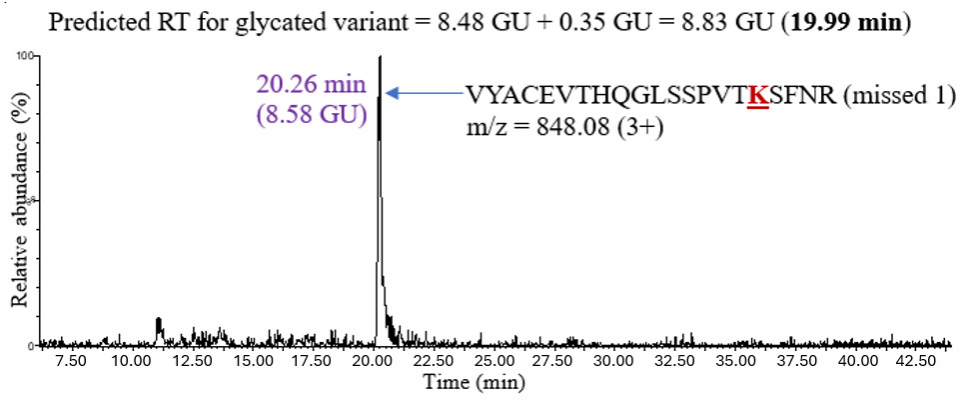
The XIC for Glycated Missed Cleavage Peptide VYACEVTHQGLSSPVTKSFNR in IgG4 (Natalizumab). The experimental retention time of the glycated variant (20.26 minutes) was close to the predicted retention time of 19.99 minutes, which was calculated using the HILIC retention coefficient for glycation.

The second unknown sample used to test the model was natalizumab, an IgG4 mAb expressed in Chinese hamster ovary (CHO) cells. The analysis proceeded similarly: predicting the retention time of a glycated peptide by adding 0.35 GU to the retention time of the unmodified peptide and confirming the chromatographic peak in the XIC. Figure 6 shows an example of glycation on peptide VYACEVTHQGLSSPVTKSFNR (missed 1) in IgG4.

The predicted retention time of the glycated peptide was 8.83 GU (19.99 minutes), and the XIC showed a chromatographic peak at 20.26 minutes, which was close to the predicted value. The retention shifts upon glycation for all the peptides observed in IgG4 are shown in Table 3. The average retention shift due to glycation in IgG4 was +0.53 GU.

**Table 3.** Prediction and characterization of glycation in the IgG4 sample (natalizumab). The experimental retention time of glycated peptides was close to the predicted value. Average retention shift upon glycation was observed to be +0.53 GU. The experimental retention times of the unmodified peptides (in minutes and in GU) are also listed for when they were detected

**IgG4 Peptide Sequence**	**Predicted RT (GU)**	**Predicted RT (min)**	**Experimental RT (min)**	**Experimental RT (GU)**	**Retention Shift upon Glycation** **(min)**	**Retention Shift upon Glycation (GU)**
VSCKASGFNIK	5.86	17.40	16.19	5.16		
VSC**K**ASGFNIK	6.21	17.93	17.67	6.03	+0.27	+0.17
ASGFNIKDTYIHWVR	5.47	16.75	17.89	6.18		
ASGFNI**K**DTYIHWVR	5.82	17.33	17.99	6.25	+1.24	+0.78
VSNKGLPSSIEK	6.75	18.67	19.74	7.82		
VSN**K**GLPSSIEK	7.10	19.08	20.01	8.40	+1.34	+0.86
GLPSSIEKTISK	6.15	17.85	18.46	6.59		
GLPSSIE**K**TISK	6.50	18.34	19.07	7.09	+1.22	+0.94
EPQVYTLPPSQEEMTKNQVSLTCLVK	8.99	19.92				
EPQVYTLPPSQEEMT**K**NQVSLTCLVK	9.34	19.62	20.32	9.59	+0.40	+0.60
LTVDKSR	5.79	17.29				
LTVD**K**SR	6.14	17.83	18.16	6.37	+0.87	+0.58
DIQMTQSPSSLSASVGDR	6.83	18.77				
**D**IQMTQSPSSLSASVGDR	7.18	19.17	19.59	7.34	+0.82	+0.51
TSQDINKYMAWYQQTPGK	7.92	19.81				
TSQDIN**K**YMAWYQQTPGK	8.27	19.96	20.16	8.30	+0.35	+0.38
YMAWYQQTPGKAPR	5.69	17.13	17.22	5.75		
YMAWYQQTPG**K**APR	6.04	17.68	17.79	6.11	+0.66	+0.42
TVAAPSVFIFPPSDEQLKSGTASVVCLLNNFYPR	4.92	14.78	15.46	4.78		
TVAAPSVFIFPPSDEQL**K**SGTASVVCLLNNFYPR	5.27	16.44	15.57	4.84	+0.79	+0.16
EAKVQWK	5.92	17.50				
EA**K**VQWK	6.27	18.02	18.70	6.77	+1.20	+0.85
VYACEVTHQGLSSPVTKSFNR	8.48	20.02				
VYACEVTHQGLSSPVT**K**SFNR	8.83	19.99	20.26	8.58	+0.24	+0.10

**Figure 7.**
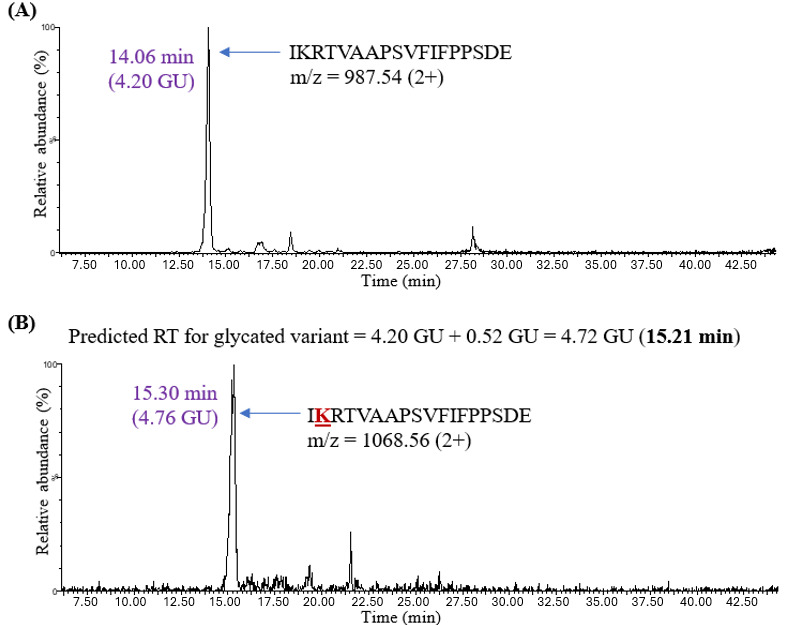
The XIC for (A) Unmodified Glu-C Peptide IKRTVAAPSVFIFPPSDE and (B) Glycated Peptide IKRTVAAPSVFIFPPSDE in Human IgG1 (Adalimumab). The experimental retention time of the glycated variant (15.30 minutes) was close to the predicted retention time of 15.21 minutes, calculated using the HILIC retention coefficient for glycation.

The HILIC behavior of glycated peptides was further investigated by evaluating peptides generated by an enzyme different from trypsin. Endoproteinase Glu-C digestion was performed on human IgG1 after in vitro glycation. Since the Badgett’s model has been described for tryptic peptides, the predicted retention time for a glycated peptide was calculated by adding 0.52 GU to the experimental retention time of its nonglycated variant (in GU) as shown in Figure 7.

The unmodified peptides in this case were present in detectable amounts since Glu-C cleaves at the C-terminus of either aspartic or glutamic acid residues with no interference from glycation at lysine residues. Peptide IKRTVAAPSVFIFPPSDE was one of the Glu-C-generated peptides observed in human IgG1 (Figure 7). The unmodified variant eluted at 14.06 minutes (4.20 GU). The predicted retention time for the glycated peptide was calculated by adding 0.52 GU to 4.20 GU, thus yielding 4.72 GU (15.21 minutes). The chromatographic peak for the glycated IKRTVAAPSVFIFPPSDE peptide (at 15.30 minutes) was noted to be very close to the predicted retention time of 15.21 minutes (Table 4). This observation indicated that the proposed model could predict retention times not only for glycated tryptic peptides but also for glycated Glu-C peptides.

**Table 4.** Glycation characterization performed on Glu-C generated peptides in human IgG1 (adalimumab). The experimental retention time of glycated peptides was observed to be close to the predicted retention time

**IgG1 Glu-C Peptide Sequence**	**Predicted RT (GU)**	**Predicted RT (min)**	**Experimental RT (min)**	**Experimental RT (GU)**
IKRTVAAPSVFIFPPSDE			14.06	4.20
I**K**RTVAAPSVFIFPPSDE	4.72	15.21	15.30	4.76
VKFNWYVDGVE			12.14	3.43
V**K**FNWYVDGVE	3.95	13.48	13.74	4.06
KSRWQQGNVFSCSVMHE			21.79	8.64
**K**SRWQQGNVFSCSVMHE	9.16	22.38	22.52	9.32
ALHNHYTQKSLSLSPG			19.89	7.38
ALHNHYTQ**K**SLSLSPG	7.90	20.72	20.97	7.89

The mAbs used in this study are pure protein samples; however, we expect that the proposed model would benefit researchers characterizing complex protein mixtures. Retention time prediction would allow one to narrow the retention time search, thereby enabling faster analyses. Such a prediction can also provide an additional level of confidence when characterizing glycated peptides.

## Conclusions

The glycated peptides were resolved from their unmodified variants using HILIC chromatography. Glycation introduces a hydrophilic glucose moiety onto the protein, resulting in a slight overall increase in HILIC retention. Several standard peptides and proteins were used to derive a retention coefficient for glycation, with a dextran ladder as the calibrant. This coefficient was incorporated into an existing peptide retention prediction model to predict and identify glycated peptides in unknown complex mAb samples, such as adalimumab (human IgG1) and natalizumab (IgG4). In all cases, the experimental retention time of glycated peptides was consistent with the predicted value. On average, a glycated peptide eluted 0.52 GU later than its unmodified variant. We propose that this value, when added to Badgett’s tryptic peptide retention model, can predict the retention time of glycated tryptic peptides. Additionally, the proposed model accurately predicts the retention time of glycated peptides generated by Glu-C enzymatic digestion of human IgG1. It is predicted that this model will aid the analysis of complex protein mixtures by providing an additional layer of confidence in peak identifications and reducing analysis time.

## References

[1] (2024). Biopharmaceuticals market size & share analysis – trends, drivers, competitive landscape, and forecasts (2024–2030).

[2] Jenkins N. (2007). Modifications of therapeutic proteins: challenges and prospects. Cytotechnology.

[3] Kaltashov I. A., Bobst C. E., Abzalimov R. R., Wang G., Baykal B., Wang S. (2012). Advances and challenges in analytical characterization of biotechnology products: mass spectrometry-based approaches to study properties and behavior of protein therapeutics. Biotechnol Adv.

[4] Ligier S., Fortin P. R., Newkirk M. M. (1998). A new antibody in rheumatoid arthritis targeting glycated IgG: IgM anti-IgG-AGE. Br J Rheumatol.

[5] Brownlee M. (1995). Advanced protein glycosylation in diabetes and aging. Annu Rev Med.

[6] Maillard L. C. (1912). Action of amino acids on sugars. Formation of melanoidins in a methodical way. Compt Rend.

[7] Popova E. A., Mironova R. S., Odjakova M. K. (2010). Non-enzymatic glycosylation and deglycating enzymes. Biotechnol Biotechnol Equip.

[8] Palimeri S., Palioura E., Diamanti-Kandarakis E. (2015). Current perspectives on the health risks associated with the consumption of advanced glycation end products: recommendations for dietary management. Diabetes Metab Syndr Obes.

[9] Videira P. A. Q., Castro-Caldas M. (2018). Linking glycation and glycosylation with inflammation and mitochondrial dysfunction in Parkinson's disease. Front Neurosci.

[10] Quan C., Alcala E., Petkovska I.. (2008). A study in glycation of a therapeutic recombinant humanized monoclonal antibody: where it is, how it got there, and how it affects charge-based behavior. Anal Biochem.

[11] Banks D. D., Hambly D. M., Scavezze J. L., Siska C. C., Stackhouse N. L., Gadgil H. S. (2009). The effect of sucrose hydrolysis on the stability of protein therapeutics during accelerated formulation studies. J Pharm Sci.

[12] Miller A.K., Hambly D.M., Kerwin B.A., Treuheit M.J., Gadgil H.S. (2011). Characterization of site-specific glycation during process development of a human therapeutic monoclonal antibody. J Pharm Sci.

[13] Kashyap S. R., Osme A., Ilchenko S.. (2018). Glycation reduces the stability of ApoAI and increases HDL dysfunction in diet-controlled type 2 diabetes. J Clin Endocrinol Metab.

[14] Zhang B., Yang Y., Yuk I.. (2008). Unveiling a glycation hot spot in a recombinant humanized monoclonal antibody. Anal Chem.

[15] Goetze A. M., Liu Y. D., Arroll T., Chu L., Flynn G. C. (2012). Rates and impact of human antibody glycation in vivo. Glycobiology.

[16] Brady L. J., Martinez T., Balland A. (2007). Characterization of nonenzymatic glycation on a monoclonal antibody. Anal Chem.

[17] Wei B., Berning K., Quan C., Zhang Y. T. (2017). Glycation of antibodies: modification, methods and potential effects on biological functions. mAbs.

[18] Lapolla A., Fedele D., Reitano R.. (2004). Enzymatic digestion and mass spectrometry in the study of advanced glycation end products/peptides. J Am Soc Mass Spectrom.

[19] Duivelshof B. L., Fekete S., Guillarme D., D'Atri V. (2019). A generic workflow for the characterization of therapeutic monoclonal antibodies—application to daratumumab. Anal Bioanal Chem.

[20] Badgett M. J., Boyes B., Orlando R. (2018). Peptide retention prediction using hydrophilic interaction liquid chromatography coupled to mass spectrometry. J Chromatogr A.

[21] Zhou Y., Priya S., Ong J. Y. (2024). Characterizing glycosylation of adeno-associated virus Serotype 9 capsid proteins generated from HEK293 cells through glycopeptide mapping and released glycan analysis. Microorganisms.

[22] Vrdoljak A., Trescec A., Benko B., Hecimovic D., Simic M. (2004). In vitro glycation of human immunoglobulin G. Clin Chim Acta.

